# Supplementary material for: Investigating the anti-osteosarcoma effects of Patchouli alcohol through protein network mapping and in vitro experiments
Source: PLoS One. 2025 Sep 24;20(9):e0332202. doi: 10.1371/journal.pone.0332202 (PMC12459782; doi:10.1371/journal.pone.0332202)

Figure 6

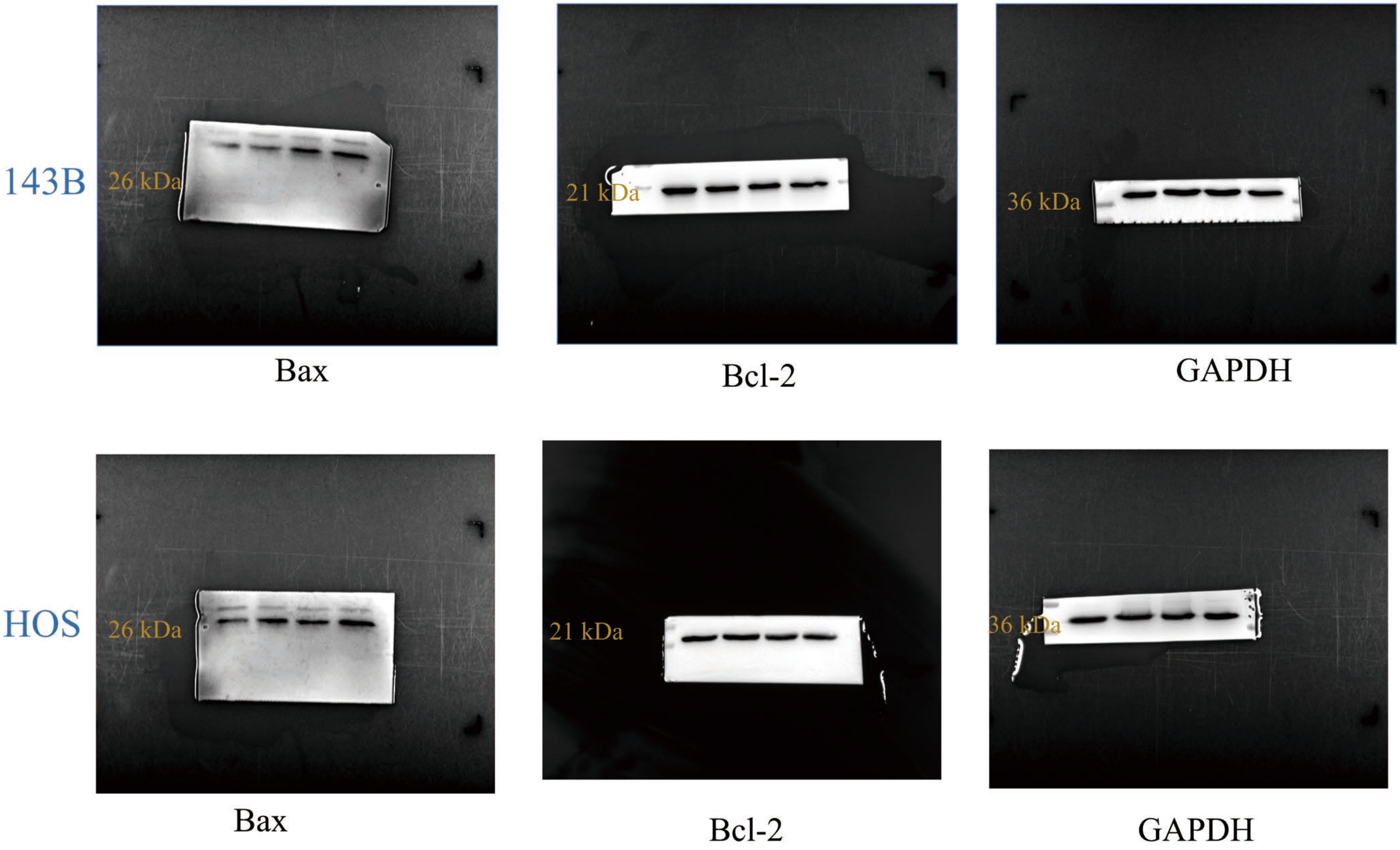

143B
26 kDa
Bax
21 kDa
Bcl-2
36 kDa
GAPDH
HOS
26 kDa
Bax
21 kDa
Bcl-2
36 kDa
GAPDH

Figure 7

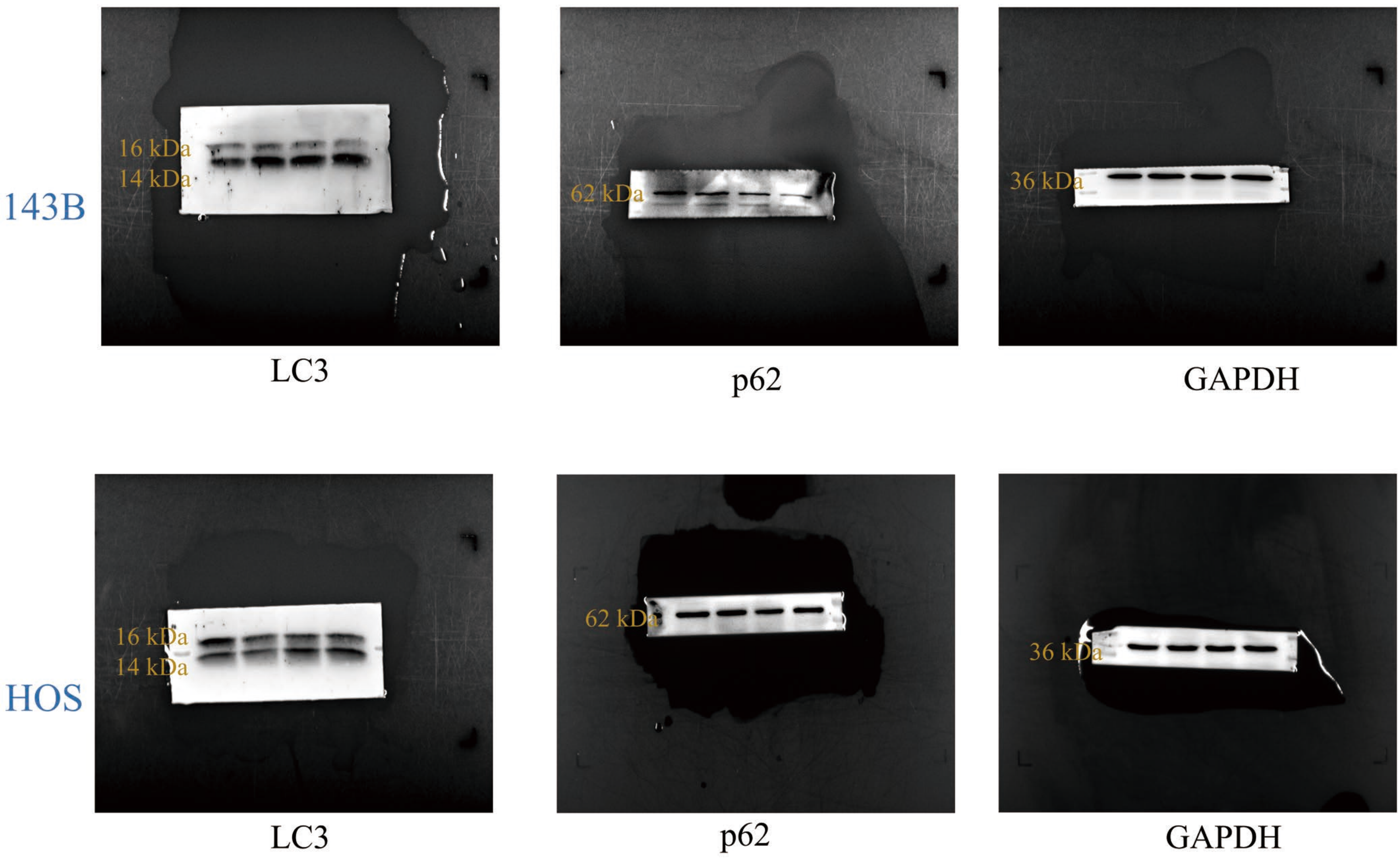
143B
16 kDa
14 kDa
LC3
62 kDa
p62
36 kDa
GAPDH
HOS
16 kDa
14 kDa
LC3
62 kDa
p62
36 kDa
GAPDH

Figure 8 143B Cell

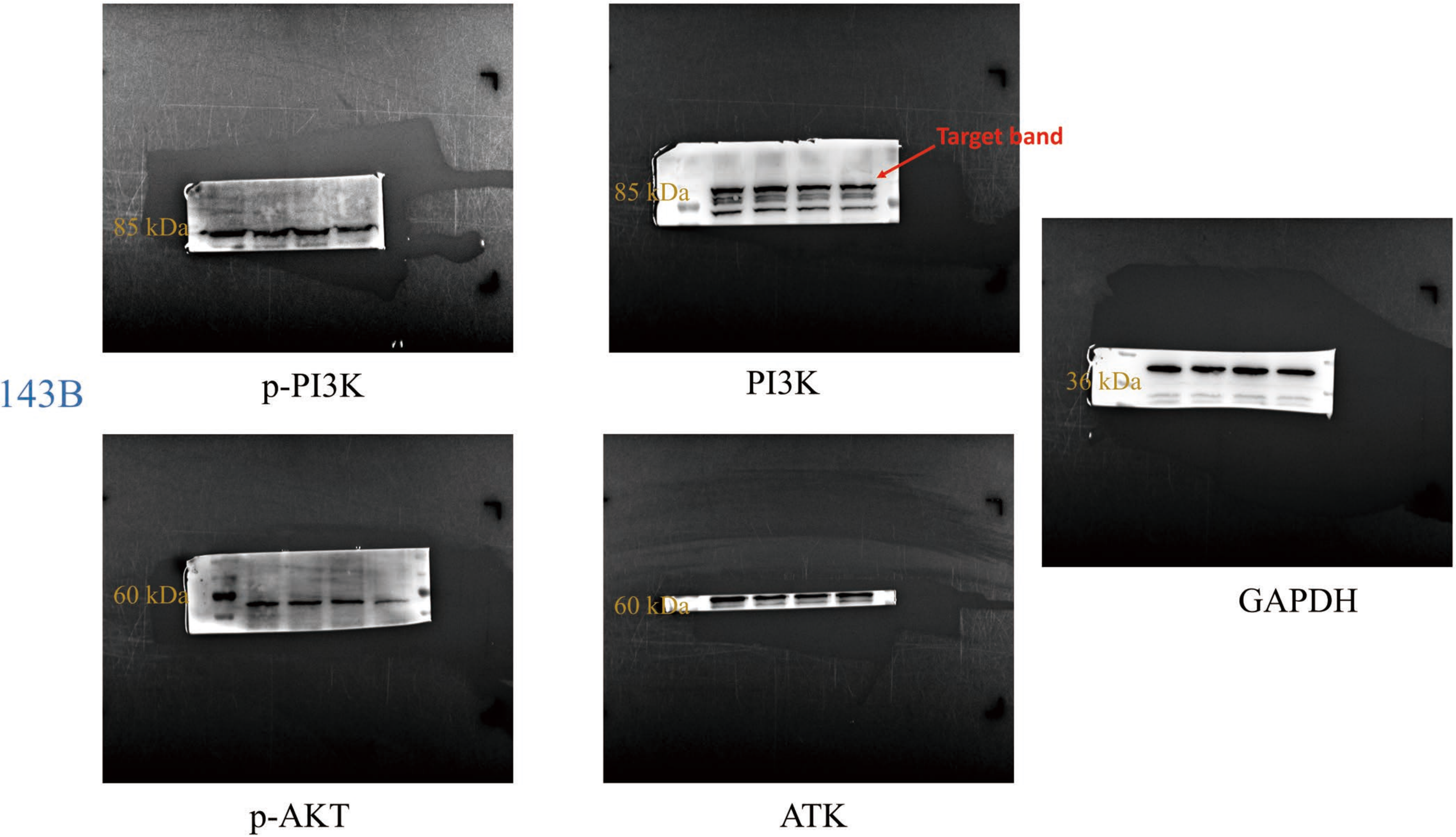

Figure 8 HOS Cell

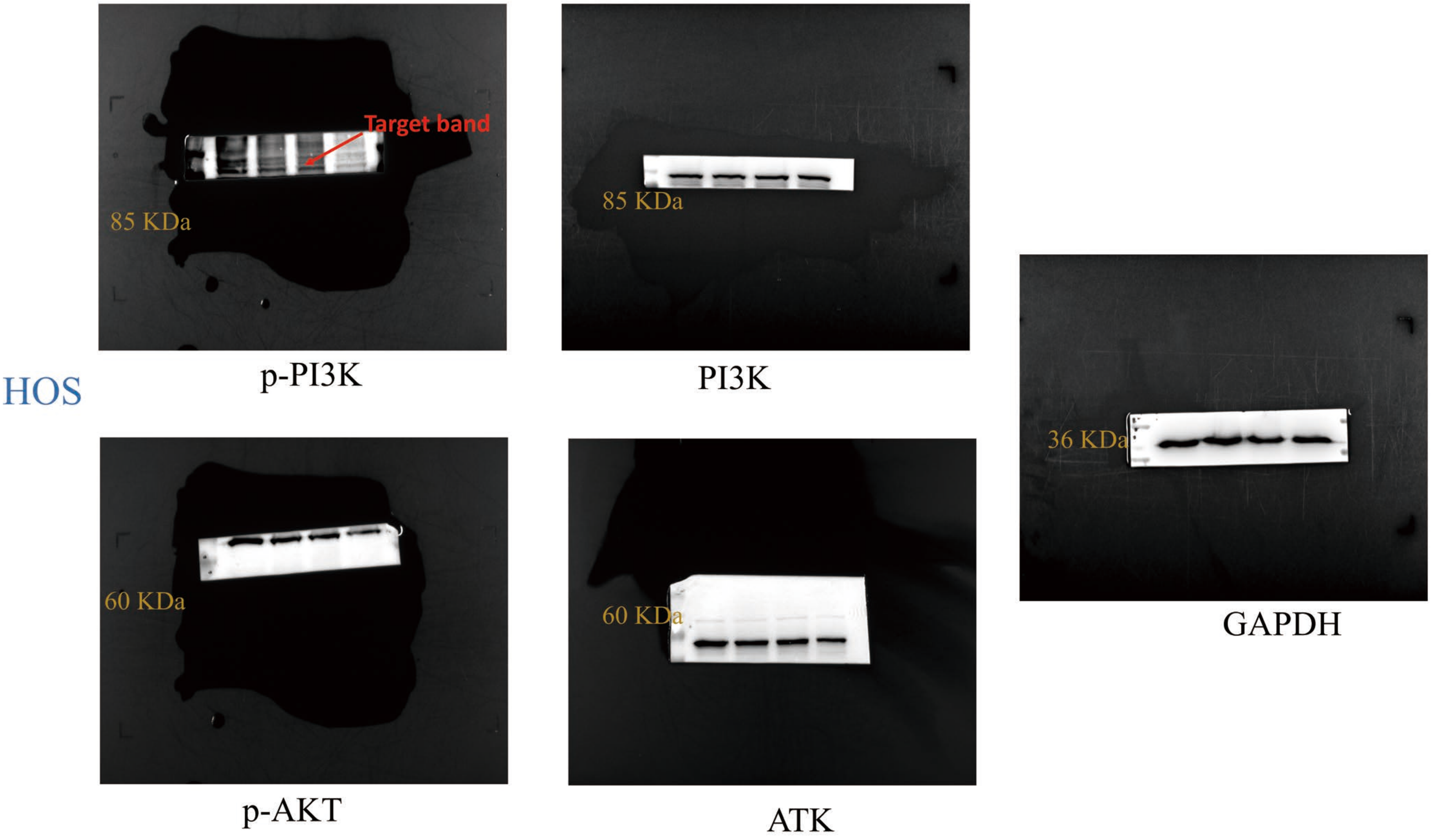

Supplement: S1 Data — (PDF) [file pone.0332202.s001.pdf]
