# Supplementary material for: Investigating the anti-osteosarcoma effects of Patchouli alcohol through protein network mapping and in vitro experiments
Source: PLoS One. 2025 Sep 24;20(9):e0332202. doi: 10.1371/journal.pone.0332202 (PMC12459782; doi:10.1371/journal.pone.0332202)
Supplement: Attachment — Submitted filename: Response Letter.docx [file pone.0332202.s003.docx]

Dear Editors and Reviewers:

Thank you for your letter and the reviewers' insightful comments regarding our manuscript, titled "*Exploration of the Anti-osteosarcoma Activity of Patchouli Alcohol Based on Network Pharmacology and In Vitro Experiments*" (ID: PONE-D-24-41403). We sincerely appreciate the reviewers’ valuable feedback, which has been instrumental in improving the quality and clarity of our work. In response to the editor's and reviewers’ suggestions, we have conducted minor revisions to our manuscript and incorporated additional content to further substantiate our findings. Additionally, we have carefully revised the manuscript to ensure full compliance with the journal’s formatting guidelines. The revised sections have been highlighted in red for easy reference. We greatly appreciate your time and consideration, and we look forward to your feedback on our revised submission.

Respond to the reviewer's comments:

***Question 1.* Title**: Most readers may not be familiar with the term "network pharmacology." I recommend reconsidering the title to make it more accessible such as *Investigating the Anti-Osteosarcoma Effects of Patchouli Alcohol through Protein Network Mapping and In Vitro Experiments*. If you prefer to retain "network pharmacology" in the title, I suggest explaining this term early in the introduction to ensure all readers understand its relevance.

**Response:** Thank you for your valuable suggestion. We understand that "network pharmacology" might be a less familiar term for some readers. We have revised the title to make it more accessible, as you suggested. The new title is: **"*****Investigating the Anti-Osteosarcoma Effects of Patchouli Alcohol through Protein Network Mapping and In Vitro Experiments.*"** We hope this revision addresses your concern and enhances the comprehensibility of our work. Thank you again for your thoughtful feedback!

***Question 2.* Cell Line Testing**: Please clarify whether the cell lines used in your experiments were tested for mycoplasma contamination, as this is an important aspect of ensuring the validity of in vitro studies.

**Response:** Thank you for highlighting this important point. We confirm that all cell lines used in our experiments were tested for mycoplasma contamination prior to the study. These tests were conducted using Myco-Lumi™ Luminescent mycoplasma detection kit (Beyotime Institute of Biotechnology; C0298S), and all results were negative, ensuring the validity and reliability of our in vitro experiments. We have added this information to the Methods section of the manuscript for clarity and transparency.

***Question 3.* Introduction:** I recommend adding a full paragraph about Patchouli Alcohol (PA) in the introduction, including details such as its half-life, route of elimination, pharmacokinetics/pharmacodynamics (PK/PD), safety, and maximum tolerated dose (MTD). This information will provide valuable context for readers.

**Response:** Thank you for your insightful suggestion. We agree that providing additional details about Patchouli Alcohol (PA) will enhance the context and understanding of our study. In response to your recommendation, we have added a dedicated paragraph in the introduction that includes information on the half-life, route of elimination, pharmacokinetics/pharmacodynamics (PK/PD), safety profile, and maximum tolerated dose (MTD) of PA. Specifically, we have incorporated relevant data from existing literature to provide a comprehensive overview of PA’s characteristics and its relevance to our investigation of its anti-osteosarcoma effects.

We believe this addition will give readers a clearer understanding of PA’s therapeutic potential and its role in our study.

***Question 4.*** **Discussion**: Additionally, please include relevant references that discuss PA, particularly in combination with other anticancer drugs. A useful resource is PubMed, where several relevant studies can be found [here](https://pubmed.ncbi.nlm.nih.gov/?term=Patchouli+alcohol+cancer&sort=date).

https://pubmed.ncbi.nlm.nih.gov/?term=Patchouli+alcohol+cancer&sort=date

**Response:** Thank you for your suggestion to include additional references discussing Patchouli Alcohol (PA), particularly in combination with other anticancer drugs. We appreciate your recommendation to explore PubMed as a resource. In response, we have reviewed relevant studies from the suggested link and identified several recent publications that discuss the anticancer effects of PA and its potential synergistic effects when used in combination with other therapeutic agents. We have incorporated these references into both the introduction and discussion sections of the manuscript to provide a more comprehensive context for PA’s role in cancer therapy.

We have made every effort to improve the manuscript and have implemented minor revisions. These changes do not alter the overall content or structure of the paper. Although we have not provided an itemized list of revisions, we have highlighted the changes in red in the revised manuscript for your convenience. We sincerely appreciate the time and effort that the editors and reviewers have dedicated to our work. We hope that the modifications will meet your approval. Should any further revisions be needed, we are more than willing to make the necessary adjustments. PLOS ONE is a journal of great renown, and we are hopeful that with these improvements, our manuscript will meet the high standards of your journal and be considered for publication. Once again, thank you very much for your valuable comments and suggestions.
