# Supplementary material for: Investigating the anti-osteosarcoma effects of Patchouli alcohol through protein network mapping and in vitro experiments
Source: PLoS One. 2025 Sep 24;20(9):e0332202. doi: 10.1371/journal.pone.0332202 (PMC12459782; doi:10.1371/journal.pone.0332202)
Supplement: Attachment — Submitted filename: Response to reviewer.docx [file pone.0332202.s004.docx]

Thank you for your letter and for the reviewers’ insightful comments on our manuscript entitled “Exploration of the Anti-osteosarcoma Activity of Patchouli Alcohol Based on Network Pharmacology and In Vitro Experiments” (ID: PONE-D-24-41403). We sincerely appreciate the reviewers’ valuable feedback, which has greatly helped us to improve both the quality and clarity of the manuscript. In response to reviewers’ suggestions, we have made minor revisions, with the updated sections highlighted in red for ease of reference. We are grateful for your time and consideration, and we look forward to your further feedback on our revised submission.

Respond to the reviewer's comments:

***Question 1.*** The term “network pharmacology” has been substituted in the title, but the keywords are still referring to network pharmacology. Also, the sequence of keywords needed to be re-considered.

**Response:** We thank the reviewer for this helpful observation. To align with the revised title and the study’s primary focus, we have removed “network pharmacology” from the keywords. We also conducted a manuscript-wide check and replaced or removed any residual mentions of “network pharmacology” where appropriate. For clarity and indexing, the remaining keywords have been reordered.

Revisions made in the manuscript:

*Previous keywords*: osteosarcoma; network pharmacology; apoptosis; Patchouli alcohol; autophagy.

*Revised keywords*: Osteosarcoma; Patchouli alcohol; Protein network mapping; Apoptosis; Autophagy.

***Question 2.***The structure formula of Patchouli alcohol (PA) is not found in this manuscript. It is better to be shown with high resolution in figure 1 or figure 3.

**Response:** We appreciate the reviewer’s valuable suggestion. In the revised version, we have supplemented the structural formula and molecular formula of Patchouli alcohol (PA) in Figure 3, presented with high resolution to ensure clarity and readability. We believe this addition will help readers more intuitively understand the compound investigated in this study. The manuscript has been revised by updating Figure 3 to incorporate the chemical structure and molecular formula of PA, and the corresponding figure legend has been adjusted accordingly.

***Question 3.*** The reason that the concentrations of PA are 25, 50 100 μM is not explained in the content. Please show some data to declare the origin of these concentrations.

**Response:** We thank the reviewer for this constructive suggestion. The concentrations of PA (25, 50, and 100 µM) were selected according to previous reports in which this range showed significant biological activity without apparent cytotoxicity, while concentrations above 100 µM exhibited toxicity. Based on these findings, we adopted 25-100 µM as a safe and effective range for evaluating the pharmacological effects of PA in our study. To clarify this rationale, we have revised the Methods section (PA treatment) to include the following statement:

“The concentrations of PA (25, 50, and 100 µM) were chosen according to previous studies indicating that this range exerts biological activity without detectable cytotoxicity, whereas concentrations above 100 µM tend to induce toxicity^1, 2^”

1.	Kumar, K. J. S.; Vani, M. G.; Chinnasamy, M.; Lin, W.-T.; Wang, S.-Y., Patchouli Alcohol: A Potent Tyrosinase Inhibitor Derived from Patchouli Essential Oil with Potential in the Development of a Skin-Lightening Agent. *Cosmetics* **2024,** *11* (2), 38.

2.	Lee, H. S.; Lee, J.; Smolensky, D.; Lee, S. H., Potential benefits of patchouli alcohol in prevention of human diseases: A mechanistic review. *International immunopharmacology* **2020,** *89* (Pt A), 107056.

***Question 4***. In figure 4C, the figure legend says “Cycle arrest of 143B and HOS cells detected by flow cytometry. **p* < 0.05 vs. control group.” But there is no data bar or * tag in the column diagram. Please check the figure and revise this mistake.

**Response:** We thank the reviewer for pointing this out. The raw data were correct; however, the original statistical presentation was not sufficiently clear. To improve clarity, we re-plotted the data for Figure 4C using a more intuitive format and added the appropriate statistical annotations. The revised figure now accurately reflects the data and is consistent with the figure legend.

***Question 5.*** This research lacks of novelty but adhere to writing conventions. To improve the quality and publishing possibility, it is necessary to address more about the creativity and significance of this research.

**Response:** We thank the reviewer for this important suggestion. In the revised manuscript, we have strengthened the Discussion to more clearly highlight the novelty and significance of this work. Specifically, we emphasize that this is the first study to investigate patchouli alcohol in osteosarcoma, where it induces both apoptosis and autophagy through suppression of PI3K/Akt signaling. By integrating Protein network mapping, molecular docking, and experimental validation, our study provides a disease-specific mechanistic framework and reveals actionable targets that may inform future therapeutic combinations. These revisions better underscore the creativity and significance of our findings.

We sincerely appreciate the time and effort that the editors and reviewers have dedicated to our work. We hope that the revisions will meet with your approval, and we remain willing to make any further adjustments if required. *PLOS ONE* is a journal of high repute, and we trust that with these improvements, our manuscript will meet its rigorous standards and be considered for publication. Once again, we thank you for your valuable comments and suggestions.
